# Evaluation of TcpF-A2-CTB Chimera and Evidence of Additive Protective Efficacy of Immunizing with TcpF and CTB in the Suckling Mouse Model of Cholera

**DOI:** 10.1371/journal.pone.0042434

**Published:** 2012-08-07

**Authors:** Gregory A. Price, Randall K. Holmes

**Affiliations:** Department of Microbiology, University of Colorado School of Medicine, Aurora, Colorado, United States of America; East Carolina University School of Medicine, United States of America

## Abstract

The secreted colonization factor, TcpF, which is produced by *Vibrio cholerae* 01 and 0139, has generated interest as a potential protective antigen in the development of a subunit vaccine against cholera. This study evaluated immunogenicity/protective efficacy of a TcpF holotoxin-like chimera (TcpF-A2-CTB) following intraperitoneal immunization compared to TcpF alone, a TcpF+CTB mixture, or CTB alone. Immunization with the TcpF-A2-CTB chimera elicited significantly greater amounts of anti-TcpF IgG than immunization with the other antigens (*P*<0.05). Protective efficacy was measured using 6-day-old pups reared from immunized dams and orogastrically challenged with a lethal dose of El Tor *V. cholerae* 01 Inaba strain N16961. Protection from death, and weight loss analysis at 24 and 48 hours post-infection demonstrated that immunization with TcpF alone was poorly protective. However, immunization with TcpF+CTB was highly protective and showed a trend toward greater protection than immunization with CTB alone (82% vs 64% survival). Immunization with the TcpF-A2-CTB chimera demonstrated less protection (50% survival) than immunization with the TcpF+CTB mixture. The TcpF-A2-CTB chimera used for this study contained the heterologous classical CTB variant whereas the El Tor CTB variant (expressed by the challenge strain) was used in the other immunization groups. For all immunization groups that received CTB, quantitative ELISA data demonstrated that the amounts of serum IgG directed against the homologous immunizing CTB antigen was statistically greater than the amount to the heterologous CTB antigen (*P*≤0.003). This finding provides a likely explanation for the poorer protection observed following immunization with the TcpF-A2-CTB chimera and the relatively high level of protection seen after immunization with homologous CTB alone. Though immunization with TcpF alone provided no protection, the additive protective effect when TcpF was combined with CTB demonstrates its possible value as a component of a multivalent subunit vaccine against *Vibrio cholerae* 01 and 0139.

## Introduction

The bacterium *Vibrio cholerae* is the etiologic agent responsible for the acute diarrheal disease cholera. There are over 200 serogroups of cholera but only 2 are known to cause epidemics: 01 and 0139. Serogroup 01 can be further subdivided into the El Tor and classical biotypes each with several serotypes. Cholera is spread by the fecal-oral route and outbreaks are caused by contamination of food and water sources due to unsanitary conditions. Prevention of cholera outbreaks can be achieved with modern sanitation and safe potable water sources [1]. However for financially strapped, impoverished countries the overhaul of their hygienic infrastructure is difficult. The WHO estimates there are at least 884 million people who lack access to safe drinking water and another 2.6 billion without proper sanitation [2]. In lieu of adequate sanitation and safe water sources, the development of efficacious vaccines to prevent cholera is an appropriate goal for endemic and at risk countries. Unfortunately the currently licensed whole-cell killed vaccines (WCK) elicit limited long-term protection necessitating the development of more effective vaccines [3].

Once ingested *V. cholerae* colonizes the small intestine where it secretes cholera toxin (CT) [4]. Cholera toxin is the primary virulence factor responsible for the profuse watery diarrhea associated with cholera. Cholera toxin is an AB_5_ toxin composed of one catalytic A polypeptide (CTA) and five identical B polypeptides (CTB) [5]. CTB is the non-toxic binding domain of CT, and it forms a donut-like structure composed of the five B polypeptides associated by non-covalent interactions. The nontoxic A2 domain of CTA passes through the central pore of CTB, tethering the A and B subunits together by non-covalent interactions [5]. CT secreted by *V. cholerae* binds to its receptor the monosialosyl ganglioside G_M1_ on the host cells [6]. The bound toxin is internalized by endocytosis and retrograde transport, and the catalytic A fragment (CTA1) is delivered to the cytosol by retrotranslocation from the endoplasmic reticulum [7]. CTA1 ADP ribosylates the α subunit of heterotrimeric stimulatory G protein (Gsα) causing activation of adenylate cyclase and a rise in intracellular adenosine-3′,5′-monophosphate (cAMP) levels. The rise in cAMP levels triggers the opening of the chloride channels resulting in an efflux of ions and water into the intestines where it is eliminated in the stool and vomitus [7].

TcpF is a secreted virulence factor of unknown function that is thought to play a role in microcolony formation in the small intestine [8]. The *tcpF* gene is part of the *tcp* operon which encodes another important virulence factor the toxin-coregulated pilus (TCP) [9]. TCP is a type IV pilus composed of the pilin subunit TcpA [10], and is absolutely required for colonization in mice and humans [11], [12]. *In vitro* expression of TCP causes the filaments to bundle to mediate bacterial autoagglutination [13]. In the infant mouse, TCP functions by mediating bacterium-to-bacterium interactions as well as mediating attachment to epithelial cells [14]. Though it has been demonstrated that TCP is necessary for TcpF secretion [15], TcpF is not required for TCP autoagglutination and may play an independent role in colonization [8]. As with TCP, TcpF has also been shown to be necessary for colonization in the infant mouse [8], [15]. Because of its importance in colonization TcpF has been examined as a potential protective antigen in the development of a vaccine against *V. cholerae* 01. Studies using passively administered hyper-immune anti-TcpF sera demonstrated protection in the infant mouse following oral challenge with lethal doses of *V. cholerae* 01 [8], [16]. It was also demonstrated that an anti-TcpF monoclonal antibody combined with a sub-protective dose of TcpA antisera provided additive protection compared to either antibody used alone [16]. The importance of TcpF for colonization in humans remains to be elucidated; however TcpF is expressed during human infections since convalescent sera from patients with cholera recognize TcpF [17]. Taken together these data suggest that TcpF is a protective antigen and may be useful in the development of a subunit vaccine against cholera.

As mentioned above, CTB is the non-toxic receptor binding domain of CT. Vaccine studies using detoxified CT or CTB in addition to other antigens have demonstrated synergistic protection against cholera in various animal models [18]–[21]. Besides acting as a protective antigen, CTB is also a potent adjuvant in mice. Numerous studies have demonstrated its ability to augment antibody responses to conjugated or co-administered antigens [22]–[26]. Detoxified CT and CTB have also been extensively studied in humans with few side-effects [27]–[30], and recombinant CTB is a component of the currently licensed oral cholera vaccine Dukoral [31].

In the current study we explored the potential of TcpF as a protective antigen when administered by itself, in combination with CTB, and with CTB in the form of a holotoxin-like chimera. Holotoxin-like chimeras are created by genetically fusing an antigen of choice in frame with the non-toxic A2 subunit of CT and co-expressing the resulting fusion as a periplasmic protein together with CTB [32], [33]. Holotoxin-like chimeras offer potential advantages as vaccines because it has been demonstrated that the immunogenicity of protein protective antigens can often be enhanced if they are physically coupled to CTB [24], [25], [34] or incorporated into holotoxin-like chimeras [35], [36]. Our results extend the study of TcpF as a protective antigen to an active immunization model and demonstrate that immune responses to TcpF and CTB can contribute additively to protection of infant mice against lethal challenge with *V. cholerae* 01 Inaba strain N16961.

## Results

### Properties of the TcpF-A2-CTB Chimera

**Figure 1:**
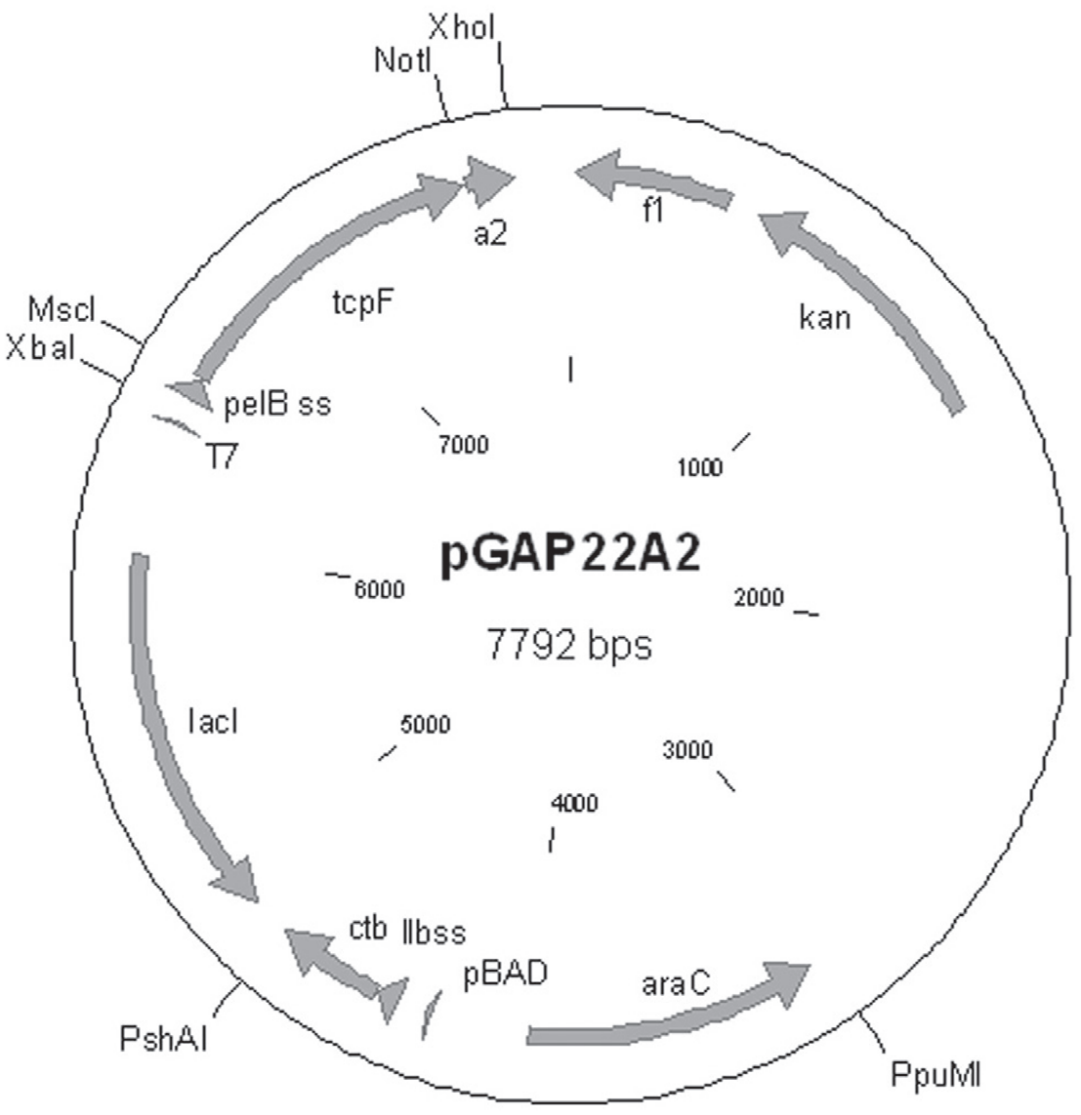
Schematic representation of the dual promoter TcpF-A2-CTB expression plasmid pGAP22A2. The IPTG inducible T7 promoter controls the mature *tcpF-a2* gene product in frame and down-stream of the *pelB* leader sequence. The arabinose inducible pBAD promotor controls the mature *ctb* gene in frame with the *ltIIB* leader sequence.

**Figure 2:**
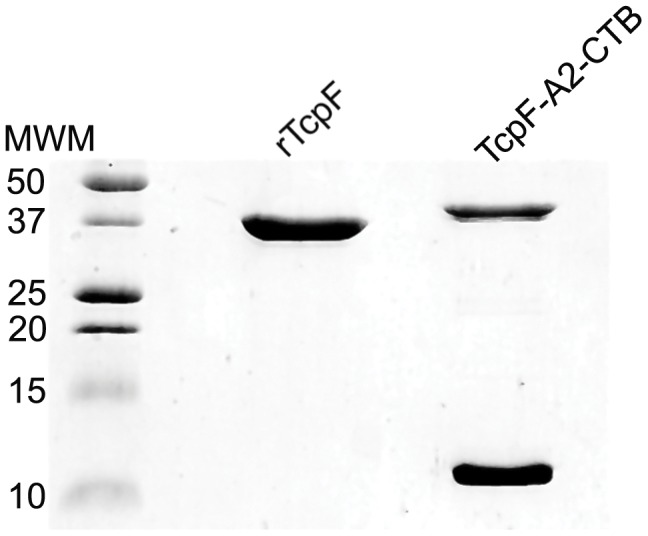
Coomasie blue stained SDS-PAGE gel of purified recombinant TcpF-A2-CTB chimera and TcpF proteins. Samples were reduced and boiled prior to loading on a 15% SDS-PAGE gel, causing the TcpF chimera to separate into the larger TcpF-A2 (∼42 kDa) fusion protein and the monomeric CTB proteins (11.5 kDa).

**Figure 3:**
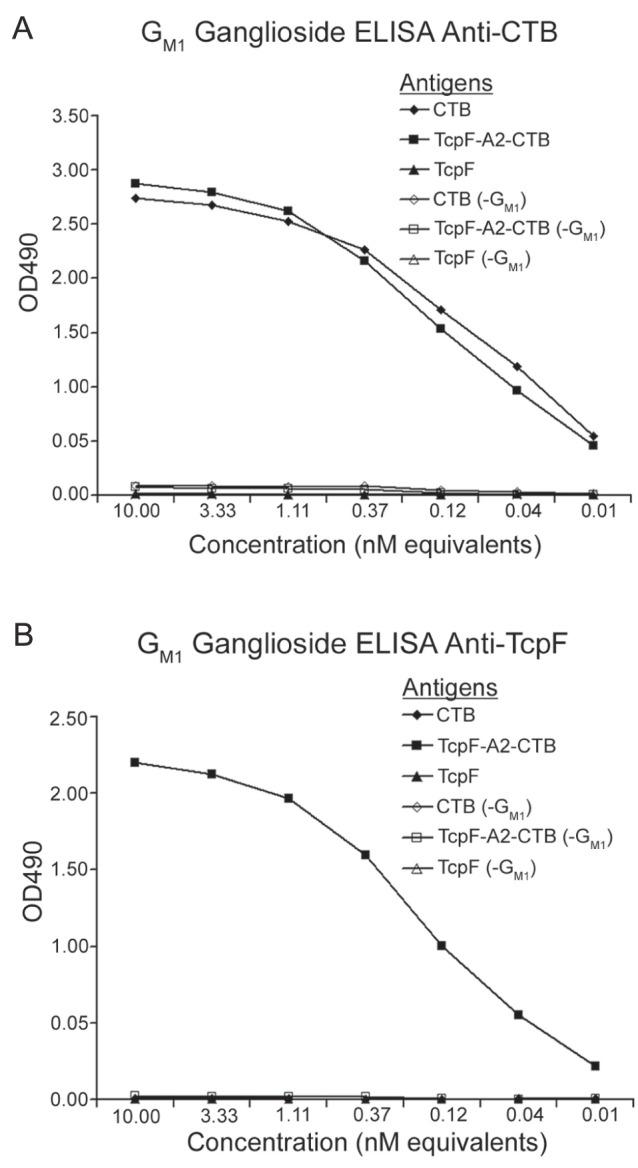
G_M1_ ganglioside ELISA demonstrates functional receptor binding by the TcpF-A2-CTB chimera and CTB. Several dilutions from stock solutions containing equimolar amounts of TcpF chimera, CTB, or TcpF were loaded onto ELISA plates coated with G_M1_ ganglioside and serially diluted. Plates were probed with either rabbit anti-CTB (A) or TcpF antibody (B) followed by secondary probing with HRP-goat anti-rabbit IgG. Plates were read at OD_490_ and optical densities were recorded. As controls duplicate antigen samples were assayed on empty plates (−G_M1_) to demonstrate that CTB binding to immobilized G_M1_ ganglioside is required to generate a signal in this assay.

The plasmid, pGAP22A2, was used to express the TcpF-A2-CTB chimera in *E. coli* (Fig. 1). The purified chimera was analyzed using SDS-PAGE, and upon heating and denaturation the chimera broke down into the TcpF-A2 fusion protein (∼42 kDa) and the CTB monomers (11.5 kDa; Fig. 2). As can be seen in Fig. 2, the TcpF-A2 fusion protein migrated slower than recombinant TcpF due to the presence of the A2 domain. In order to demonstrate the tethering of TcpF to CTB via the A2 subunit and functional binding of the CTB domain to its cognate receptor G_M1_ ganglioside, we performed G_M1_ ganglioside ELISAs. As can be seen in Fig. 3A, both the TcpF-A2-CTB chimera and CTB bound avidly to G_M1_ ganglioside coated ELISA plates as shown by binding to an anti-CTB antibody. Only minor background binding was seen on plates not containing G_M1_ ganglioside. ELISA plates were also probed with an anti-TcpF antibody to demonstrate that the chimera did indeed contain the TcpF domain and that TcpF by itself did not bind to GM1 ganglioside. As demonstrated in Fig. 3B only the TcpF-A2-CTB chimera reacted with the anti-TcpF antibody in the G_M1_ ganglioside ELISAs.

### Serum and Fecal Antibody Responses Following Intraperitoneal Immunization

**Figure 4:**
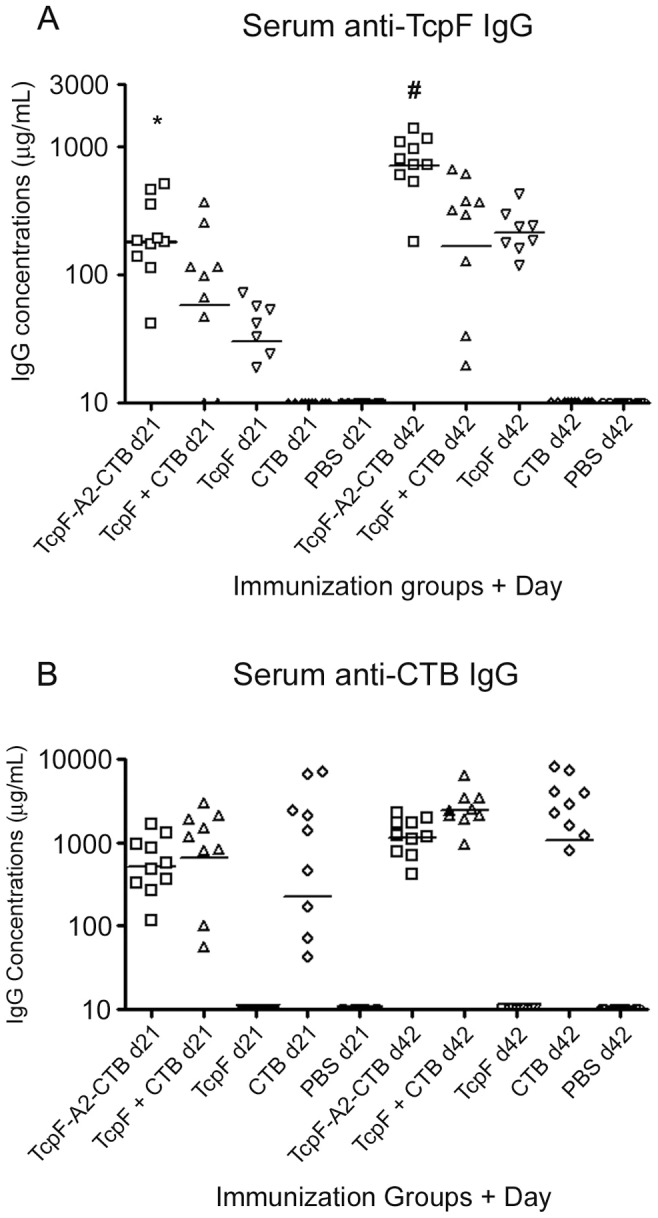
Quantitative ELISA analysis of serum samples. Day 21 and 42 serum samples were assayed for anti-TcpF (A) or anti-CTB (B) IgG amounts. Each data point represents an individual mouse within a group and the horizontal bars indicate the geometric mean of each group. Statistical differences between groups were analyzed using ANOVA with the Tukey-Kramer post-test analysis (*P<0.05 versus TcpF+CTB, and *P*<0.001 versus TcpF; ^#^ *P*<0.001 versus TcpF+CTB, and *P*<0.01 versus TcpF).

Five groups of 8–10 female CD-1 mice were immunized IP three times at 14 day intervals (days 0, 14, and 28) for this study. In order to keep the amounts of the antigenic components comparable in the immunization regimens, the TcpF-A2-CTB chimera was delivered at 50 µg/dose and the other immunization groups were given equimolar amounts of TcpF, CTB, or both. Blood and fecal samples were collected at days −1, 21, and 42, and analyzed for antigen-specific antibody amounts using quantitative ELISA (qELISA). Serum antibody responses to TcpF were greater following immunization with the TcpF-A2-CTB chimera and were significantly greater both at day 21 and 42 than the responses to TcpF alone or to TcpF mixed with CTB (Fig. 4A; *P*<0.05). The mean anti-TcpF antibody titers elicited by immunization with TcpF alone or TcpF mixed with CTB were not significantly different either at day 21 or day 42 (Fig. 4A; *P*>0.05). Antibody responses to CTB were robust in all groups containing CTB regardless of the presence of TcpF and no significant differences were observed on either day 21 or day 42 between these groups (Fig. 4B; *P*>0.05).

**Figure 5:**
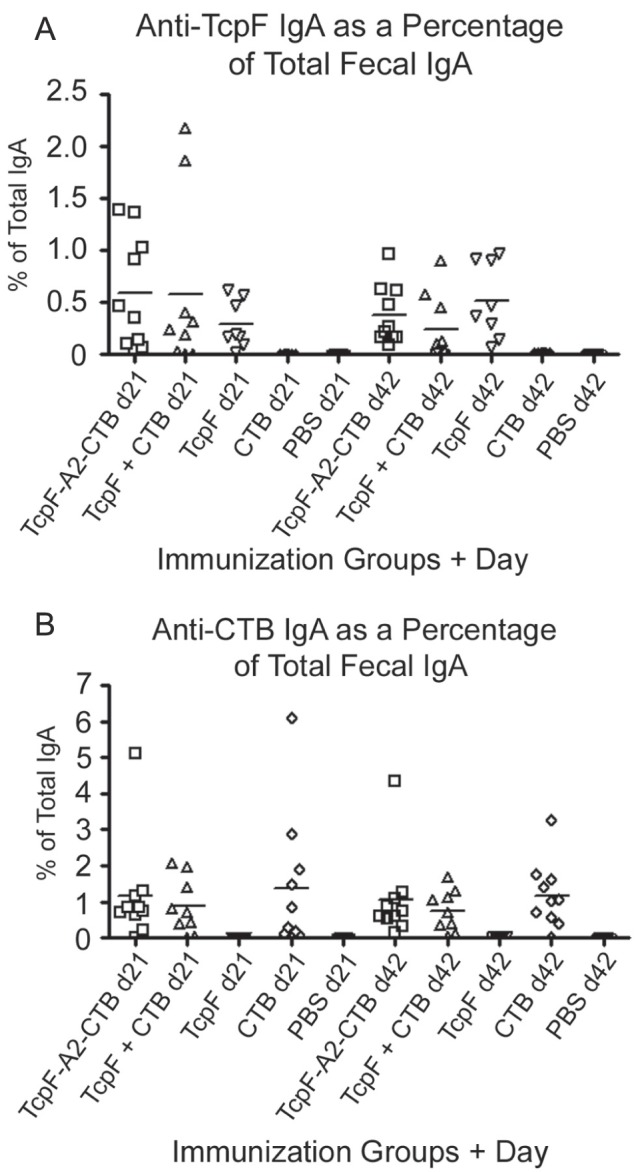
Anti-TcpF and anti-CTB amounts as percentages of total fecal IgA. Fecal extracts from day 21 and 42 were assayed using quantitative ELISA for anti-TcpF (A) or anti-CTB (B) IgA. In order to normalize the data total IgA levels in each extract were also determined using quantitative ELISA. The data presented in the graphs represent the percentage specific IgA antibody amounts to the total IgA amounts in each sample. Each data point represents one mouse and the horizontal bars represent the geometric mean of each group.

Fecal antigen-specific IgA responses and total IgA levels were measured in fecal extracts using qELISA. The levels of specific TcpF and CTB antibodies in Fig. 5 are expressed as the percentage of antigen-specific IgA to total IgA in each fecal extract. This normalization procedure was used in an effort to limit differences based on mouse-to-mouse and sample-to-sample variations. No TcpF- or CTB-specific IgA was detectable in the fecal samples collected before immunization. As can be seen in Fig. 5, IP immunization was able to induce antigen-specific fecal IgA responses to both TcpF and CTB. However, unlike the enhanced TcpF-specific serum IgG responses elicited by the chimera, the TcpF-specific fecal IgA responses elicited by TcpF alone, by the TcpF+CTB mixture, and by the TcpF-A2-CTB chimera were not significantly different either on day 21 or day 42 (*P*>0.05). In terms of geometric mean levels, fecal CTB-specific IgA responses were higher than those obtained against TcpF and did not differ significantly between the CTB only, TcpF+CTB, and TcpF-A2-CTB chimera groups (Fig. 5; *P*>0.05).

### Suckling Mouse Challenge

We utilized the cholera suckling mouse challenge model in an effort to determine whether the antigens used for immunization here could confer protection against a lethal dose of *V. cholerae* 01. Immunized females were mated for 15 days starting on day 43 (one day following their last blood/fecal collection), and subsequently their six day old reared pups were orogastrically inoculated with 15 LD_50_ of *V. cholerae* 01 Inaba strain N16961 and monitored for survival over a 48 hour period. As assay controls, additional pups were sham infected with media only. As can be seen in Table 1, the group with the highest protective efficacy, 82% survival rate, was from dams immunized with TcpF+CTB. The group with the second highest survival rate was from dams immunized with CTB only (67%), but this level was not significantly different from the group immunized with TcpF+CTB (*P* = 0.17). Interestingly, challenged pups from mothers immunized with the TcpF-A2-CTB chimera had a significantly lower % survival than the pups from mothers immunized with TcpF+CTB (50% versus 82%, *P* = 0.002; Table 1). This was a surprising result as serum anti-TcpF IgG titers were significantly higher in the TcpF-A2-CTB chimera immunization group than the TcpF+CTB immunization group. (Fig. 4A; *P*<0.001). Also, the TcpF-A2-CTB chimera immunization group did not have as high a survival rate as the CTB only immunization group (50% versus 67%; Table 1), although this difference did not reach statistical significance (*P*>0.05). Among all of the immunized groups, pups from mothers immunized with TcpF only had the poorest protection from death (17% survival) which was not statistically different from the PBS control group which had 100% lethality (*P*>0.05).

**Table 1:** Infant Mouse Challenge (15LD_50_ *V. cholerae* N16961).

Immunization	Challenge	Survivors/Total (% Survival)
Various	AKI medium*	19/19 (100%)%
PBS	*V. cholerae* N16961	0/20 (0%)
TcpF-A2-CTB	*V. cholerae* N16961	17/34 (50%)∧
TcpF+CTB	*V. cholerae* N16961	42/51 (82%)#
TcpF	*V. cholerae* N16961	7/41 (17%)
CTB	*V. cholerae* N16961	20/30 (67%)@

* Infant pups were from a mixture of immunization groups and challenged with AKI media only.

% Significantly different from all other groups (*P*<0.05) except TcpF+CTB (*P* = 0.1).

# Significantly different from TcpF chimera group (*P* = 0.002) and TcpF group (*P*<0.0001).

@Significantly different from TcpF group (P<0.0001).

∧ Significantly different from TcpF group (*P* = 0.003).

### Measurement of Weight Loss at 24 and 48 Hours Post Infection

**Figure 6:**
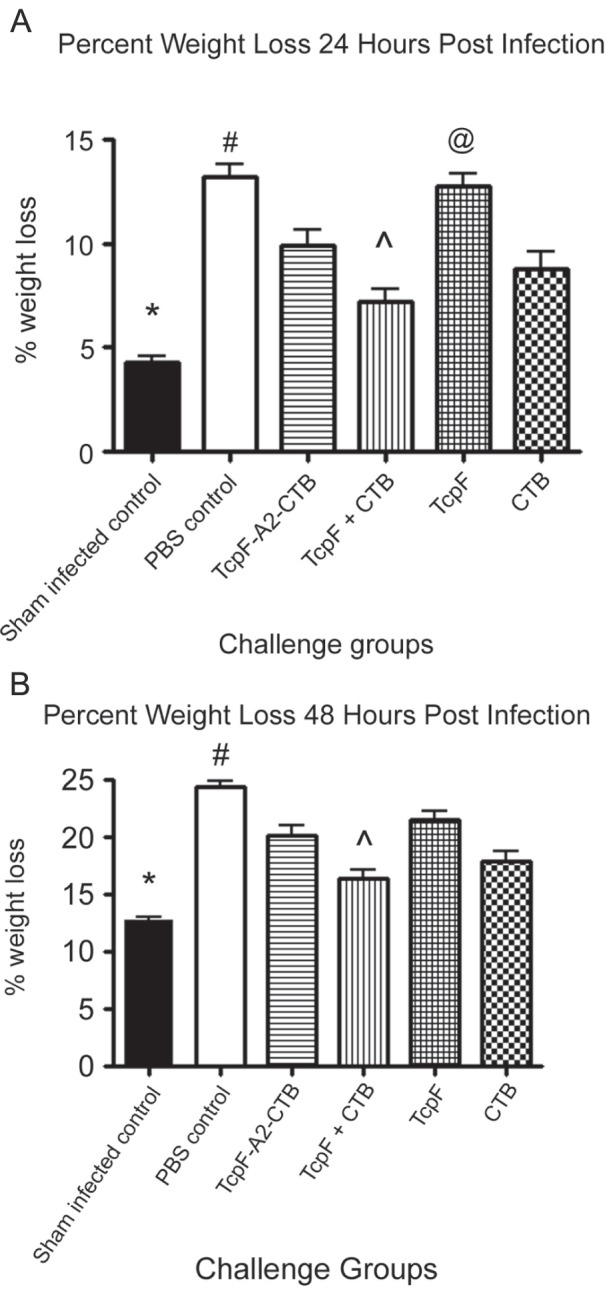
Average weight loss of pups at 24 and 48 hours post-infection with *V. cholerae*. Pups were weighed immediately before, and 24 and 48 hours post-infection with 15LD_50_ of *V. cholerae*. Weight losses at 24 hours (A) and 48 hours (B) were compared to their initial weights at time  = 0. Error bars represent the SEM. Statistical analysis was performed using ANOVA followed by the Tukey-Kramer post-test. Symbols 7A, *statistically different from all groups (*P*<0.05) except TcpF+CTB (*P*>0.05); ^#^statistically different from all groups groups (*P*<0.05) expect TcpF (*P*>0.05); ^∧^statistically different from TcpF chimera and TcpF only groups (*P*<0.05); ^@^statistically different from all groups (*P*<0.05) except the PBS group (*P*>0.05). Symbols 7B, *statistically different from all groups (*P*<0.05); ^#^statistically different from TcpF+CTB and CTB only groups (*P*<0.001); ^∧^statistically different from all goups (*P*<0.05) except the CTB only group (*P*>0.05).

As with humans, infant mice develop severe diarrhea during infection with *V. cholerae* 01 [37]. In an effort to assess the severity of the diarrhea during the course of the infection by a non-invasive method, we measured the weight of each pup immediately before challenge and at 24 and 48 hours post-challenge, and we compared the results with similar measurements on pups challenged with *V. cholerae* or sham-inoculated with AKI medium. At 24 hours there was a dramatic difference in weight loss between the sham infected and the non-immune PBS immunized controls, demonstrating that the *V. cholerae* inoculum was virulent (Fig. 6A). The weight loss observed in the sham infected control group was significantly less than in all the infected groups (*P*<0.05) except for the group immunized with TcpF+CTB (*P*>0.05). The PBS immunized group had significantly greater weight loss in comparison with all groups except the TcpF immunized group which demonstrated little protective efficacy. The TcpF-A2-CTB chimera immunized group did have significantly greater weight loss than the group immunized with TcpF+CTB (*P*<0.05), consistent with the lower protective efficacy of immunization with TcpF-A2-CTB. By 48 hours all infected groups had significantly greater weight losses than the sham infected controls (*P*<0.05). Only the groups immunized with TcpF+CTB and CTB alone had weight losses that were significantly less than the PBS immunized group (Fig. 6B; *P*<0.05).

### Immunological Differences between El Tor and Classical CTB Genotypes

**Figure 7:**
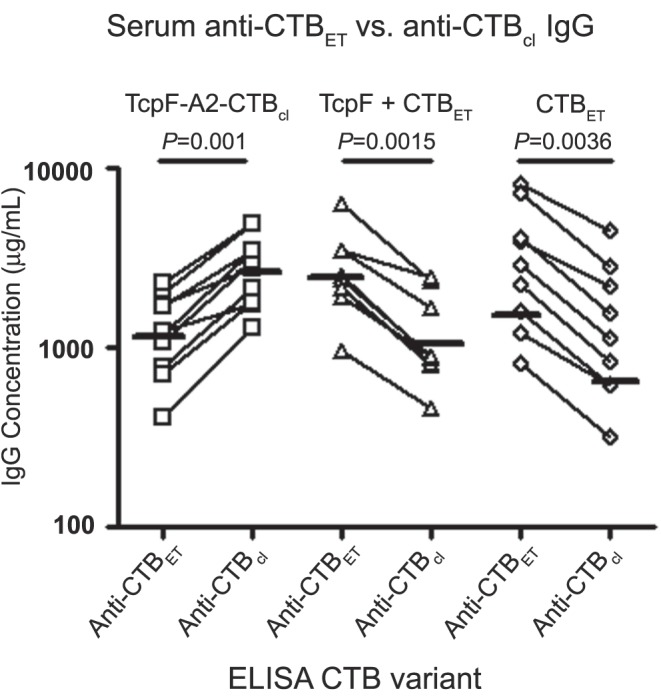
Quantitative measurements of serum IgG antibodies specific for CTB_ET_ or CTB_cl_ in mice immunized with TcpF-A2-CTB_cl_ chimera, TcpF+CTB_ET_, or CTB_ET_. Day 42 serum samples were assayed for anti-CTB IgG amounts to either CTB_ET_ or CTB_cl_. Each data point represents the anti-CTB IgG amount for an individual mouse. Anti-CTB IgG amounts specific for CTB_ET_ and CTB_cl_ in each individual mouse are connected with a line. Horizontal lines per group represent the geometric mean titer to either CTB_ET_ or CTB_cl_. Above each grouping are the antigens that were used for immunization and the corresponding *P*-value obtained for within group comparisons. Statistical differences were analyzed using a two-tailed paired t-test, and *P*-values less than 0.05 were considered significant.

Since our TcpF-A2-CTB chimera gave poorer protection than the TcpF+CTB admixture and CTB alone, we evaluated the consequences of using the classical CTB variant in our chimera and the El Tor CTB variant for the other immunization regimens. To this end, we compared the serum IgG anti-CTB levels achieved by each immunization regimen described above by using qELISA assays with purified recombinant classical CTB (CTB_cl_) and El Tor CTB (CTB_ET_). Though CTB_cl_ and CTB_ET_ differ by only 2 amino acids [38], every mouse had a lower anti-CTB IgG level against the heterologous CTB variant than against the homologous CTB variant. The mean amounts of specific IgG antibody against the homologous variant of CTB in each immunization group were greater by more than 2-fold than against the heterologous CTB variant, and these levels were statistically significant in each group (Fig. 7; *P*≤0.003).

## Discussion

Previous studies reported that orogastric administration of rabbit antisera, purified rabbit polyclonal antibodies or mouse monoclonal antibodies directed against TcpF conferred protection against challenge with several different classical or El Tor strains of *V. cholerae* 01 in the infant mouse model of cholera [8], [16]. In preliminary experiments for this study, we prepared a TcpF-specific rabbit antiserum against the TcpF protein from El Tor *V. cholerae* 01 Inaba strain N16961 that exhibited a high titer against TcpF in ELISA assays. Somewhat surprisingly, we found that orogastric administration of this hyperimmune anti-TcpF antiserum failed to confer protection against challenge with *V. cholerae* 01 Inaba strain N16961 in the infant mouse model of cholera (data not shown). These divergent results between reported studies and our findings could reflect differences among the anti-TcpF sera prepared in different rabbits with respect to the total amounts of TcpF-specific antibodies or the relative amounts of antibodies directed against protective or non-protective epitopes of TcpF. They could also reflect differences in characteristics of the *V. cholerae* 01 stocks prepared in different laboratories for use as challenges in the infant mouse model of cholera. Systemic analysis of such possible variables will require additional experiments in future studies.

In additional preliminary experiments, we immunized adult CD-1 mice with TcpF by the orogastric route at doses up to 200 **µ**g at two-week intervals up to 4 times, but demonstrated only weak to no TcpF-specific serum antibody responses (data not shown). Other investigators recently showed that IP immunization of adult female mice with outer membrane vesicles prepared from *V. cholerae* 01 resulted in excellent protection for their reared pups against intestinal colonization by *V. cholerae* 01 after orogastric challenge; the protection appeared to be mediated primarily by IgG antibodies, since no antigen-specific IgA antibodies were detected in stomach contents from the pups [39], [40]. These findings influenced our choice of IP immunization as a promising regimen for eliciting strong TcpF-specific antibody responses and facilitating our evaluation of the contribution of TcpF-specific antibodies to protective immunity in the infant mouse model of cholera.

In the current study, we demonstrated that intraperitoneal immunization of dams with TcpF alone was ineffective at eliciting protective immunity following subsequent challenge of the pups with 15 LD_50_ doses of El Tor *V. cholerae* 01 Inaba strain N16961 in the suckling mouse model of cholera. We used a large challenge dose (15 LD_50_) because our preliminary experiments demonstrated that a high level of protection could be achieved by immunization with CTB alone. This experimental design allowed us to demonstrate that immunization with TcpF plus CTB elicited a likely additive protective effect that might not have been demonstrable with use of a smaller challenge dose. Although active immunization of dams with TcpF alone did not protect pups against the high 15 LD_50_ challenge dose used in our study, it has not yet been determined if immunization with TcpF alone in this experimental model could protect pups against lower challenge doses of *V. cholerae*. Intraperitoneal immunization of dams with CTB alone elicited much higher levels of antigen-specific serum IgG and provided much greater protection against challenge with *V. cholerae* 01 than immunization with TcpF did. Our study clearly demonstrates the importance of CT neutralization in protection both from death and from weight loss caused by diarrhea in the infant mouse model of cholera.

In terms of potential relevance for vaccine development, several studies have demonstrated that direct conjugation of antigens to CTB or incorporation into holotoxin-like chimeras can improve the immune response to the antigen of interest compared with admixtures of the same antigen with CTB [24], [25], [34]–[36]. Two approaches have been employed for direct conjugation, chemical and genetic. Chemical conjugation has the disadvantages of being labor intensive, which would add to the cost of producing a vaccine, and non-specific, which could result in variability between vaccine preparations and cause problems for vaccine standardization and regulatory approval. Utilizing a genetic approach as with our TcpF-A2-CTB chimera, only one antigen must be produced and purified to create a bivalent and homologous vaccine antigen. Studies that demonstrated enhanced immunogenicity of antigens attached to CTB or incorporated into holotoxin-like chimeras focused mostly on mucosal vaccination approaches [24], [25], [34]–[36]. Our study demonstrates that enhancement of immunogenicity can also occur using chimeric antigens for immunization via a parenteral route. This is a potentially important consideration for vaccine development because less antigen and fewer immunizations could likely be used to achieve protective immunity.

Despite eliciting statistically higher anti-TcpF antibody titers (*P*<0.05) and similar anti-CTB titers, immunization with the TcpF-A2-CTB chimera did not protect as well from death and weight loss in the infant mouse model as immunization with a mixture of TcpF+CTB. It is possible that fusion of TcpF to the A2 subunit of CT had adverse effects on important and potentially protective TcpF epitopes. If this were the case however, we would still have expected to see protective efficacy and weight losses in the group immunized with the TcpF-A2-CTB chimera similar to those in the groups immunized with CTB alone.

One possible explanation for the lower protective efficacy of our TcpF-A2-CTB chimera is that it contains the classical variant of CTB that is heterologous to the CTB produced by the El Tor *V. cholerae* N16961 challenge strain, instead of the homologous El Tor CTB variant used in our CTB alone and TcpF+CTB mixture immunization groups. Although these two CTB variants differ by only 2 amino acids [38], it was striking that our qELISA data showed 2.2-fold greater mean concentrations of IgG antibody in the serum of the immunized mice that bound to the homologous vs. the heterologous CTB variant. Within-group statistical analyses demonstrated these levels to be significantly different (*P*≤0.003). This suggests that the amino acids that differ between the El Tor and classical variants of CTB affect immunodominant protective epitopes that have a significant impact on toxin neutralization. A previous study characterized a monoclonal antibody against classical CT that did not recognize the El Tor variant of CT produced by *V. cholerae* N16961 [41]. Furthermore hyperimmune sera against classical CT were not as efficient at neutralizing an El Tor variant of CT [42]. To the best of our knowledge, however, this is the first report that shows quantitative differences in the amounts of CTB-specific IgG antibodies in mouse polyclonal anti-CTB antisera that exhibit specific binding to homologous and heterologous variants of CTB. To date, there are six known genotypes of CTB [43], and hybrid El Tor strains containing the classical CTB variant have been emerging recently throughout the world [44]–[47]. Whether the emergence of these hybrid strains could be driven in part by selective pressure due to immune evasion or adaptation that permits better survival of the hybrid strains in the intestinal tract in host populations with a high prevalence of immunity to the previously more prevalent El Tor strains *of V. cholerae* remains to be elucidated. However the data presented here indicate that it may be desirable or necessary to include multiple CTB variants in the formulation of a broadly protective subunit vaccine against cholera.

Although protection afforded by immunization with TcpF alone was poor and immunization with CTB alone was substantial, we were able to demonstrate a trend toward additive protective effects of immunization with TcpF+CTB against death following a high challenge dose (15 LD_50_) of *V. cholerae* 01 El Tor N16961. Though protection from death with TcpF+CTB immunization showed only a trend toward greater protection from death than immunization with CTB alone, weight loss analysis at 24 hours showed that the TcpF+CTB immunization group was the only immunization group that did not exhibit weight loss significantly greater than that of sham infected controls (*P*>0.05). Furthermore, a previous study showed that a TcpF monoclonal antibody combined with a sub-protective dose of a TcpA polyclonal antibody conferred additive protection compared to either antibody alone when administered passively in the infant mouse model [16]. Therefore TcpF may have value as one protective antigen in the development of a multivalent subunit vaccine that could achieve a high degree of protective efficacy against cholera. This concept was demonstrated during the development of the acellular pertussis vaccine where a five-component formulation elicited better protective efficacy in humans than a two-component vaccine [48]. Future studies are warranted to assess the potential contributions of protective antigens such as CTB, TcpA, TcpF, and other candidate antigens for overall protective efficacy against cholera in the process of developing subunit vaccines against *V. cholerae* 01 and 0139.

## Materials and Methods

### Ethics Statement

All procedures involving immunization and breeding of adult CD1 mice and challenge of pups with *Vibrio cholerae* N16961 in the infant mouse model of cholera were approved by the University of Colorado Denver Animal Care and Use Committee. The studies were done under protocol 33701206(10)F which was initially approved on 10/11/2006 and protocol 33709(11)1E which was initially approved on 11/4/2009. This Institution has an Animal Welfare Assurance of file with the Office of laboratory Animal Welfare. The Assurance number is PHS A3269-01 (09/31/2011). This Institution is accredited by the Association for Assessment and Accreditation of Laboratory Animal Care (AAALAC) - File Number 00235.

### Construction of Recombinant Expression Plasmids

Genomic DNA from the El Tor *Vibrio cholerae* strain N16961 was used for amplification of the *tcpF* and *ctb* genes and the *cta2* coding region. All PCR amplified gene products were first subcloned into TOPO pCR2.1 (Invitrogen, Grand Island, NY) and sequenced prior to expression vector assembly. For the construction of the TcpF chimera the *tcpF* gene was PCR amplified using the forward primer oTcpF-Fmsc, and the reverse primer oTcpF-Rnot containing the restriction sites MscI and NotI respectively (See Table 2 for sequences). The coding region for the *a2* gene was also PCR amplified using the forward primer oA2-Fnot, and the reverse primer oA2-Rxho, containing the restriction sites NotI and XhoI respectively (Table 1). The forward primer oA2-Fnot contained a point mutation in the *a2* coding sequence resulting in replacement of a cysteine residue by a serine (Table 2; point mutation underlined). The *tcpF* and *a2* genes were ligated into pET22b(+)[EMD Biosciences, Gibbstown, NJ ]; *tcpF* inserted into the MscI and NotI sites and the *a2* into the NotI and XhoI sites. Both *tcp*F and *a2* were in frame with each other, and the *tcpF* gene was inserted in frame with the *pelB* signal sequence found on pET22b(+). This created the expression vector pGAP19. An arabinose inducible CTB_cl_ expression plasmid pARCTB2 was used as a template to amplify the *araC* gene, the *pBAD* promotor and the mature *ctb_cl_* in frame with the *E. coli ltIIB* signal sequence. Primers oAraC-FppuMI and oCTB-RpshAI were used for PCR amplification and contained the restriction sites PpuMI and PshAI respectively. Following confirmation of correct sequences the amplicon was restriction digested with the above flanking restriction sites and subcloned into pGAP19. This created the T7 and pBAD dual expression vector pGAP21A2. In order to create a kanamycin resistant variant of pGAP21A2, the kanamycin resistant expression vector pET28b(+) (EMD Biosciences, Gibbstown, NJ) was restriction digested with EcoRI and PpuMI. This fragment containing the *f1* origin and the kanamycin resistance cassette was isolated and inserted into the EcoRI and PpuMI site of pGAP21, an early version of pGAP21A2. This kanamycin resistant vector backbone was used for the assembly of the kanamycin resistant TcpF chimera plasmid pGAP22A2 (Fig. 1). For expression of the TcpF chimera pGAP22A2 was used in this study.

**Table 2:** Primers used for making expression constructs.

Primers	Sequence
oTcpF-Fmsc	ACT**TGGCCA**CATTTAATGATAATTATAGTTC
oTcpF-Rnot	T**GCGGCCGC**TTTAAAGTTCTCTGAATATG
oA2-Fnot	T**GCGGCCGC**AAGTAATACTAGCGATGAAAA
oA2-Rxho	T**CTCGAG**TCATAATTCATCCTTAAT
oAraC-FppuMI	TAC**GGGTCCT**GACATCTTTGTGGACACATC
oCTB-RpshAI	CT**GACTATCGTC**TTAATTTGCCATACTAATTG
oTcpF-Fnde	A**CATATG**GCATTTAATGATAATTATAGTTC
oTcpF-Rxho	CTTATCT**CTCGAG**TTTAAAGTTCTCTGAATATGC
oCTB-Fmsc	CGC**TGGCCA**CACCTCAAAATATTACTG
oCTB-Rxho	TTT**CTCGAG**TTAATTTGCCATACTAATTGC

The TcpF expression vector was constructed by amplifying the mature *tcpF* gene using the primers oTcpF-Fnde and oTcpF-Rxho containing the Nde and Xho restriction sites respectively (Table 2). Following confirmation of sequence the *tcpF* gene was inserted into the NdeI and XhoI sites of pET22b(+), creating the TcpF expression vector pGAP14.

The CTB_ET_ expression vector pGAP20K was constructed by PCR amplifying the mature *ctb* gene using the primers oCTB-Fmsc and oCTB-Rxho containing the MscI and XhoI restriction sites respectively. Following sequence confirmation the *ctb* gene was ligated into pET22b(+) creating the expression plasmid pGAP20. To create a kanamycin resistant expression plasmid, pGAP20 was restriction digested with XbaI and XhoI and the fragment containing the ribosome binding site plus the *pelB* signal sequence and the mature *ctb* gene was ligated into pET28b(+) creating pGAP20K. The CTB_cl_ expression vector was created using the same primers as above. Genomic DNA from the classical strain 0395 was used as a template and following sequence confirmation the classical CTB variant was inserted into pGAP20 using the MscI and XhoI restriction sites. This created the expression vector pGAP20_cl_. The new CTB_cl_ expression plasmid pGAP20_cl_ was sequenced to confirm replacement of CTB_ET_ with CTB_cl_.

### Protein Expression and Purification

All recombinant proteins were expressed under the same conditions with the exception of expression strains and antibiotic concentrations. The TcpF-A2-CTB chimera and CTB variants were expressed in *E. coli* BL-21 (DE3) cells. Recombinant C-terminal his-tagged TcpF was expressed in *E. coli* Origami™ B (DE3) cells (EMD Biosciences, Gibbstown, NJ). The TcpF chimera and CTB variants were expressed in half-liter cultures containing TCYM pH 7.5 (Tryptone 1%, NaCl 0.5%, yeast extract 0.5%, casamino acids 0.1%, MgSO_4_ 0.2%) and 100 µg/ml kanamycin. TcpF was grown in half liter cultures of TCYM pH 7.5 as well containing 100 µg/mL carbenicillin, 15 µg/mL kanamycin and 12.5 µg/mL tetracycline. Cultures were grown at 37°C with shaking at 250 rpm until they reached an optical density at 600 nm of ∼3.0. Cultures were then grown at room temperature (∼22°C) with shaking at 250 rpm for 30 min to 1 hour and then induced with 0.1 mM IPTG. Arabinose at 0.1% was also added to induce production of the TcpF-A2-CTB chimera. The cultures were put back at room temperature with shaking at 250 rpm for overnight induction (∼16–18 hours). Following induction the cells were harvested by centrifugation, and the pellets were kept at −80°C until use.

The primary purification protocol was the same for all proteins using Talon metal-affinity resin (Clontech, Mountain View, CA). Only TcpF contained a C-terminal six-histidine tag. Both CTB and TcpF-A2-CTB were efficiently purified using the Talon metal-affinity resin due to the inherent ability of CTB to bind to metal-affinity resin [49]. Pellets were thawed on ice and suspended in 50 mM NaH_2_PO_4_, 300 mM NaCl, pH 8.0. To obtain soluble extracts 2% Elugent (EMD Biosciences, Gibbstown, NJ), 50 µg/mL lysozyme, and protease inhibitors (Sigma, St. Louis, MO) were added and incubated with mixing at 4°C for 1 hour. The extract was then sonicated on ice to decrease viscosity. Any remaining insoluble material was removed by centrifugation at 25,132×g for 15 minutes at 4°C. Talon affinity resin was added to the soluble extract and incubated at room temp with shaking for 30 minutes. Following binding the Talon resin was washed with 75 bed volumes of the above phosphate buffer. Protein was eluted from the column with 250 mM imidazole in the above phosphate buffer.

Secondary ion-exchange purification steps were performed with all proteins to remove detectable amounts of contaminating proteins. The TcpF-A2-CTB chimera and recombinant TcpF were dialyzed against 40 mM Tris-Cl pH 8.1 overnight at 4°C. The dialyzed proteins were then passed through a 0.45 µM syringe filter to remove any precipitated material. Both the TcpF-A2-CTB chimera and TcpF were then purified using the anion-exchange POROS® 20 HQ resin (Applied Biosystems, Carlsbad, CA). The bound protein was eluted from the column using a linear gradient from 0 to 0.5 M NaCl in 40 mM Tris-Cl buffer at pH 8.1. The purified TcpF-A2-CTB chimera was dialyzed overnight at 4°C against 50 mM Tris buffer containing 200 mM NaCl, and 1 mM EDTA pH 7.5. Purified TcpF was dialyzed against 1x PBS overnight at 4°C.

Following the primary purification step, CTB was dialyzed against 25 mM potassium phosphate buffer pH 6.6 overnight at 4°C. The dialyzed protein was subjected to centrifugation to remove precipitated material and passed through 0.45 µM syringe filter. Ion-exchange chromatography was conducted using the strong cation-exchange resin POROS® 20 HS (Applied Biosystems, Carlsbad, CA). The bound protein was eluted from the column using a linear gradient from 0 to 0.5 M NaCl in 25 mM potassium phosphate buffer at pH 6.6. The purified CTB was dialyzed against 1x PBS overnight at 4°C. All proteins purified proteins were filter sterilized using a 0.2 µM syringe filter and stored at −80°C.

### Immunizations and Sample Collections

Female CD-1 mice 6–8 weeks old were purchased from Charles River Labs and allowed food and water ad libitum. Immunization groups consisted of 8–10 mice, and all mice were immunized intraperitoneally at 14 day intervals (day 0, 14, and 28). The group immunized with the TcpF chimera was given a dose of 50 µg of chimera per dose, and all other groups were immunized with equimolar amounts of antigen based on the amount administered to the TcpF chimera group. Blood and fecal samples were collected one day prior to the primary immunization (day -1) and at days 21 and 42 post-immunization. Blood was collected by submandibular bleeding using sterile 5 mm Goldenrod Animal Lancets (Medipoint, Inc., Mineola, NY). Fecal samples were collected from individual mice and resuspended at 5–10 µl/mg of feces in 1X PBS pH 7.4 containing 50 mM EDTA, 0.1 mg/mL soybean trypsin inhibitor, and 100 mM PMSF. Fecal pellets were vortexed until fully macerated, the insoluble material was pelleted by centrifugation, and the supernatants were removed and stored at −80°C. All animal procedures were approved by the University of Colorado Denver Institutional Animal Care and Use Committee.

### Quantitative and G_M1_ Ganglioside ELISAs

Serum and fecal extracts were analyzed for antigen-specific antibodies by performing quantitative ELISAs (qELISA) [25]. Fecal extracts were also analyzed for total IgA in each sample. To measure antigen-specific antibody levels, TcpF or CTB for use as a capture antigen was diluted to 1 µg/mL in either carbonate buffer (0.015 M Na_2_CO_3_, 0.035 M NaHCO_3_, pH 9.6) or borate buffered saline (0.05 M boric acid, 0.0012 M sodium tetraborate decahydrate, 0.1 M NaCl, pH 8.2), respectively. Goat anti-mouse IgG or goat anti-mouse IgA (Bethyl Laboratories, Montgomery, TX) for use as a capture antibody was diluted to 1 µg/mL in the above carbonate buffer. For each antigen or capture antibody, 100 µl samples were added to each well of a 96 well plate, and the plate was placed at 4°C overnight to permit antigen coating. After coating, each plate was washed two times with wash buffer (1X PBS pH 7.4 containing 0.05% Tween 20), followed by treatment for 1 hour at 37°C with blocking buffer (1x PBS pH 7.4, 5% horse serum, 0.05% Tween 20). Samples of serum or fecal extract to be tested were diluted in blocking buffer to appropriate starting concentrations, and 100 µl samples were added to the first well in duplicate rows on the assay plate and serially diluted. Plates coated with TcpF or CTB were used for assays to measure antigen-specific IgG in serum samples or antigen-specific IgA in fecal extracts, and plates coated with goat anti-mouse IgG or IgA were used for assays to measure total IgG or IgA in the samples, respectively. In addition, a mouse reference serum (Bethyl Laboratories, Montgomery, TX) with known concentrations of IgG and IgA immunoglobulins was diluted appropriately in blocking buffer and tested on plates coated with goat anti-mouse IgG and goat anti-mouse IgA, respectively, to generate standard curves for IgG or IgA. Plates were then placed at 4°C for overnight incubation. Plates were washed three times with the above wash buffer and goat anti-mouse peroxidase-conjugated antibodies (Sigma, St.Louis, MO) diluted in blocking buffer were used to detect standard curve and antigen-specific antibodies. Plates were incubated 4 hours at room temperature then washed three times with wash buffer. Plates were developed with Sigmafast™ OPD substrate (Sigma, St. Louis MO) in the dark for 30 minutes. After 30 minutes the reaction was stopped by the addition of 3 M HCl, and the plates were read at 490 nm using a Bio-Tek® Synergy Ht microplate reader (Winooski, VT). For serum samples, concentrations of anti-TcpF- or anti-CTB-specific IgG were interpolated from the IgG standard curve using KC4 v3.4 software (Bio-Tek®, Winooski, VT) and expressed in µg/ml. For fecal extracts, amounts of anti-TcpF- or anti-CTB-specific IgA were expressed as percentages of total IgA. All samples were run in duplicate per plate at 2 different dilutions, and also assayed twice on separate plates. All samples had intra and inter plate percent coefficients of variation of less than 20 (%CV<20).

For the G_M1_ ganglioside ELISAs, G_M1_ ganglioside was diluted to 1.5 nM in the above carbonate buffer and added to individual wells of a 96 well plate. The plates were put at 4°C overnight to allow for coating. Following coating the plates were washed two times with wash buffer and blocked using blocking buffer for one hour at 37°C. Equimolar amounts of antigens were diluted in blocking buffer and added in duplicate to the 96 well plates. Samples were serially diluted and placed at 30°C for one hour. Plates were washed three times and probed with either rabbit anti-TcpF or anti-CTB antisera diluted in blocking buffer for one hour at room temperature. Plates were washed again three times and goat anti-rabbit IgG HRP-conjugated antibody was diluted appropriately in blocking buffer and added to the plate. Plates were again incubated for one hour at room temperature and washed again three times. The plates were developed as above until sufficient color developed then the reaction was stopped by the addition of 3 M HCl. Plates were then read at 490 nm and the optical density was determined for each well. Each sample was also tested side-by-side with control plates not containing G_M1_ ganglioside to demonstrate receptor-specific binding to solid phase G_M1_ ganglioside.

### Infant Mouse Challenges

In preliminary experiments, we determined the LD_50_ of *V. cholerae* N16961 by inoculating 6 day old pups of outbred CD1 mice orogastrically in groups of 10 with inocula ranging from 4×10^5^ to 6.4×10^7^ CFUs in half-log increments. After infection, the pups were monitored for survival over a 48 hour time period. The LD_50_ of *V. cholerae* strain N16961 in our laboratory, calculated by the method by Reed and Muench [50], was ∼7×10^5^ CFU. To prepare the inoculum for the experiments reported here, a freezer stock of *Vibrio cholerae* N16961 was streaked out on nutrient agar plates and incubated at 30°C for 22–24 hours. Single colonies were inoculated into 40 ml samples of AKI broth (1.5% Bacto-peptone, 0.4% yeast extract, 0.5% NaCl, 0.3% NaHCO_3_) in sterile 50 mL conical tubes, and the tubes were incubated statically at 30°C for 16 hrs. The bacteria were collected by centrifugation at 7650×g for 10 minutes at room temperature. The supernatant was removed and the bacterial pellet was suspended in fresh AKI broth to an OD_600_ of 0.34, which corresponds to ∼1.1×10^9^ CFU/mL. The inoculum was prepared by diluting this stock into AKI media containing 0.01% Evan’s blue dye to a concentration predicted to contain 15 LD_50_ per 100 µl. Samples from this inoculum were plated onto LB agar plates and incubated at 30°C overnight to confirm that the viable counts were close to the predicted values.

Mouse pups were obtained from females that had been mated 1-to-1 with 11 week old male CD-1 mice for 15 days. After 15 days the males were removed and the females monitored for birth. Six day old pups were removed from their mothers for 3 hours prior to challenge and kept warm on a 30°C warming pad. Immediately prior to inoculation each pup was weighed to one hundredth of a gram and numbered on the back with a permanent marker for identification. For intragastric inoculation a 1CC syringe fitted with a 23 gauge needed and covered by 1 inch of PE50 tubing was used. Each pup was intragastrically gavaged with 100 µl of the 15 LD_50_ *V. cholerae* inoculum, or 100 µl of AKI broth alone for the sham infected group. The addition of the Evan’s blue dye to the inoculum allowed for direct visualization of stomach deposition. Following inoculation pups were housed in large Petri dishes (100×25 mm) containing sawdust bedding and placed on a 30°C warming pad. The pups were monitored for survival over the course of 48 hours and were weighed at 24 and 48 hours post-infection. Pups that succumbed to the infection prior to the 24 hour time point were weighed and the carcass included into the 24 hour time point. Those pups that died after the 24 hour time point but prior to the 48 hour endpoint were weighed and the carcass weight was included in the 48 hour endpoint. We found that carcass weights remained relatively static over the course of six hours (data not shown).

### Statistical Analysis

Statistical analyses were performed using GraphPad PRISM® 4 software. Statistical differences between groups for antigen-specific antibody amounts and percent weight losses were analyzed using ANOVA with the Tukey-Kramer post-test. Statistical differences within groups for CTB-specific antibody levels (CTB_ET_ versus CTB_cl_) were analyzed using a paired two-tailed t-test. Statistical comparisons between groups for protection from death in the infant mouse model were performed using contingency tables and *P* values were obtained using Fisher’s exact test. A *P* value less than 0.05 was considered significant.

## References

[1] Sack DA, Sack RB, Nair GB, Siddique AK (2004) Cholera. Lancet 363: 223–233. 14738797 10.1016/s0140-6736(03)15328-7

[2] WHO/UNICEF (2010) Status and progress towards the MDG target. In:Progress on sanitation and drinking water: 2010 update.: WHO Press. 6–13 p.

[3] Sinclair D, Abba K, Zaman K, Qadri F, Graves PM (2011) Oral vaccines for preventing cholera. Cochrane Database Syst Rev. CD008603. 21412922 10.1002/14651858.CD008603.pub2 PMC6532691

[4] Plotkin SA, Orenstein WA, Offit PA (2008) Vaccines. Philadelphia: Saunders/Elsevier. xvii, 1725 p.

[5] Zhang RG, Scott DL, Westbrook ML, Nance S, Spangler BD, et al (1995) The three-dimensional crystal structure of cholera toxin. J Mol Biol 251: 563–573. 7658473 10.1006/jmbi.1995.0456

[6] Holmgren J, Lonnroth I, Svennerholm L (1973) Fixation and inactivation of cholera toxin by GM1 ganglioside. Scand J Infect Dis 5: 77–78. 4199382 10.3109/inf.1973.5.issue-1.15

[7] De Haan L, Hirst TR (2004) Cholera toxin: a paradigm for multi-functional engagement of cellular mechanisms (Review). Mol Membr Biol 21: 77–92. 15204437 10.1080/09687680410001663267

[8] Kirn TJ, Taylor RK (2005) TcpF is a soluble colonization factor and protective antigen secreted by El Tor and classical O1 and O139 *Vibrio cholerae* serogroups. Infect Immun 73: 4461–4470. 16040956 10.1128/IAI.73.8.4461-4470.2005 PMC1201224

[9] Manning PA (1997) The tcp gene cluster of *Vibrio cholerae* . Gene 192: 63–70. 9224875 10.1016/s0378-1119(97)00036-x

[10] Craig L, Taylor RK, Pique ME, Adair BD, Arvai AS, et al (2003) Type IV pilin structure and assembly: X-ray and EM analyses of *Vibrio cholerae* toxin-coregulated pilus and *Pseudomonas aeruginosa* PAK pilin. Mol Cell 11: 1139–1150. 12769840 10.1016/s1097-2765(03)00170-9

[11] Herrington DA, Hall RH, Losonsky G, Mekalanos JJ, Taylor RK, et al (1988) Toxin, toxin-coregulated pili, and the toxR regulon are essential for *Vibrio cholerae* pathogenesis in humans. J Exp Med 168: 1487–1492. 2902187 10.1084/jem.168.4.1487 PMC2189073

[12] Taylor RK, Miller VL, Furlong DB, Mekalanos JJ (1987) Use of phoA gene fusions to identify a pilus colonization factor coordinately regulated with cholera toxin. Proc Natl Acad Sci U S A 84: 2833–2837. 2883655 10.1073/pnas.84.9.2833 PMC304754

[13] Kirn TJ, Lafferty MJ, Sandoe CM, Taylor RK (2000) Delineation of pilin domains required for bacterial association into microcolonies and intestinal colonization by *Vibrio cholerae* . Mol Microbiol 35: 896–910. 10692166 10.1046/j.1365-2958.2000.01764.x

[14] Krebs SJ, Taylor RK (2011) Protection and attachment of *Vibrio cholerae* mediated by the toxin-coregulated pilus in the infant mouse model. J Bacteriol 193: 5260–5270. 21804008 10.1128/JB.00378-11 PMC3187450

[15] Kirn TJ, Bose N, Taylor RK (2003) Secretion of a soluble colonization factor by the TCP type 4 pilus biogenesis pathway in *Vibrio cholerae* . Mol Microbiol 49: 81–92. 12823812 10.1046/j.1365-2958.2003.03546.x

[16] Megli CJ, Yuen AS, Kolappan S, Richardson MR, Dharmasena MN, et al (2011) Crystal structure of the *Vibrio cholerae* colonization factor TcpF and identification of a functional immunogenic site. J Mol Biol 409: 146–158. 21440558 10.1016/j.jmb.2011.03.027 PMC3098003

[17] Hang L, John M, Asaduzzaman M, Bridges EA, Vanderspurt C, et al (2003) Use of in vivo-induced antigen technology (IVIAT) to identify genes uniquely expressed during human infection with *Vibrio cholerae* . Proc Natl Acad Sci U S A 100: 8508–8513. 12826608 10.1073/pnas.1431769100 PMC166259

[18] Holmgren J, Svennerholm AM, Lonnroth I, Fall-Persson M, Markman B, et al (1977) Development of improved cholera vaccine based on subunit toxoid. Nature 269: 602–604. 72361 10.1038/269602a0

[19] Kundu J, Mazumder R, Srivastava R, Srivastava BS (2009) Intranasal immunization with recombinant toxin-coregulated pilus and cholera toxin B subunit protects rabbits against *Vibrio cholerae* O1 challenge. FEMS Immunol Med Microbiol 56: 179–184. 19453752 10.1111/j.1574-695X.2009.00563.x

[20] Pierce NF, Cray WC Jr, Sacci JB Jr (1982) Oral immunization of dogs with purified cholera toxin, crude cholera toxin, or B subunit: evidence for synergistic protection by antitoxic and antibacterial mechanisms. Infect Immun 37: 687–694. 6889574 10.1128/iai.37.2.687-694.1982 PMC347586

[21] Srivastava R, Sinha VB, Srivastava BS (1979) Re-evaluation of antibacterial and antitoxin immunities in experimental cholera. Indian J Med Res 70: 369–373. 535945

[22] Isaka M, Yasuda Y, Kozuka S, Miura Y, Taniguchi T, et al (1998) Systemic and mucosal immune responses of mice to aluminium-adsorbed or aluminium-non-adsorbed tetanus toxoid administered intranasally with recombinant cholera toxin B subunit. Vaccine 16: 1620–1626. 9713937 10.1016/s0264-410x(98)00066-8

[23] Isaka M, Yasuda Y, Mizokami M, Kozuka S, Taniguchi T, et al (2001) Mucosal immunization against hepatitis B virus by intranasal co-administration of recombinant hepatitis B surface antigen and recombinant cholera toxin B subunit as an adjuvant. Vaccine 19: 1460–1466. 11163669 10.1016/s0264-410x(00)00348-0

[24] McKenzie SJ, Halsey JF (1984) Cholera toxin B subunit as a carrier protein to stimulate a mucosal immune response. J Immunol 133: 1818–1824. 6470484

[25] Price GA, Russell MW, Cornelissen CN (2005) Intranasal administration of recombinant *Neisseria gonorrhoeae* transferrin binding proteins A and B conjugated to the cholera toxin B subunit induces systemic and vaginal antibodies in mice. Infect Immun 73: 3945–3953. 15972481 10.1128/IAI.73.7.3945-3953.2005 PMC1168620

[26] Wu HY, Russell MW (1998) Induction of mucosal and systemic immune responses by intranasal immunization using recombinant cholera toxin B subunit as an adjuvant. Vaccine 16: 286–292. 9607044 10.1016/s0264-410x(97)00168-0

[27] Bergquist C, Johansson EL, Lagergard T, Holmgren J, Rudin A (1997) Intranasal vaccination of humans with recombinant cholera toxin B subunit induces systemic and local antibody responses in the upper respiratory tract and the vagina. Infect Immun 65: 2676–2684. 9199436 10.1128/iai.65.7.2676-2684.1997 PMC175378

[28] Curlin GT, R Levine, K. M. A Aziz, A. S. M Rahman, W. F Verwey (1975) Field trial of cholera toxoid, p. 314–335. Proceedings of the Eleventh Joint Conference of the US-Japan Cooperative Medical Science Program: Department of Health, Education, and Welfare, Washington, D. C.

[29] Levine MM, Nalin DR, Craig JP, Hoover D, Bergquist EJ, et al (1979) Immunity of cholera in man: relative role of antibacterial versus antitoxic immunity. Trans R Soc Trop Med Hyg 73: 3–9. 442179 10.1016/0035-9203(79)90119-6

[30] Noriki H (1976) Evaluation of toxoid field trial in the Philippines, p. 302–310. *In* H Fukumi and Y Zimaka (ed), Proceedings of the 12th Joint Conference of the US-Japan Cooperative Medical Science Program: National Institute of Japan, Tokyo, Japan.

[31] WHO (2010) Cholera vaccines: WHO position paper. Wkly Epidemiol Rec 85: 117–128. 20349546

[32] Hajishengallis G, Hollingshead SK, Koga T, Russell MW (1995) Mucosal immunization with a bacterial protein antigen genetically coupled to cholera toxin A2/B subunits. J Immunol 154: 4322–4332. 7722290

[33] Jobling MG, Holmes RK (1992) Fusion proteins containing the A2 domain of cholera toxin assemble with B polypeptides of cholera toxin to form immunoreactive and functional holotoxin-like chimeras. Infect Immun 60: 4915–4924. 1399002 10.1128/iai.60.11.4915-4924.1992 PMC258248

[34] Rask C, Fredriksson M, Lindblad M, Czerkinsky C, Holmgren J (2000) Mucosal and systemic antibody responses after peroral or intranasal immunization: effects of conjugation to enterotoxin B subunits and/or of co-administration with free toxin as adjuvant. Apmis 108: 178–186. 10752686 10.1034/j.1600-0463.2000.d01-42.x

[35] Li X, Erbe JL, Lockatell CV, Johnson DE, Jobling MG, et al (2004) Use of translational fusion of the MrpH fimbrial adhesin-binding domain with the cholera toxin A2 domain, coexpressed with the cholera toxin B subunit, as an intranasal vaccine to prevent experimental urinary tract infection by *Proteus mirabilis* . Infect Immun 72: 7306–7310. 15557656 10.1128/IAI.72.12.7306-7310.2004 PMC529142

[36] Sultan F, Jin LL, Jobling MG, Holmes RK, Stanley SL Jr (1998) Mucosal immunogenicity of a holotoxin-like molecule containing the serine-rich *Entamoeba histolytica* protein (SREHP) fused to the A2 domain of cholera toxin. Infect Immun 66: 462–468. 9453596 10.1128/iai.66.2.462-468.1998 PMC107928

[37] Ujiiye A, Nakatomi M, Utsunomiya A, Mitsui K, Sogame S, et al (1968) Experimental Cholera in Mice I. First Report on the Oral Infection. Tropical Medicine 10: 65–71.

[38] Goel AK, Jiang SC (2010) Genetic determinants of virulence, antibiogram and altered biotype among the *Vibrio cholerae* O1 isolates from different cholera outbreaks in India. Infect Genet Evol 10: 815–819. 19580888 10.1016/j.meegid.2009.06.022

[39] Schild S, Nelson EJ, Bishop AL, Camilli A (2009) Characterization of *Vibrio cholerae* outer membrane vesicles as a candidate vaccine for cholera. Infect Immun 77: 472–484. 19001078 10.1128/IAI.01139-08 PMC2612262

[40] Schild S, Nelson EJ, Camilli A (2008) Immunization with *Vibrio cholerae* outer membrane vesicles induces protective immunity in mice. Infect Immun 76: 4554–4563. 18678672 10.1128/IAI.00532-08 PMC2546833

[41] Tamplin ML, Ahmed MK, Jalali R, Colwell RR (1989) Variation in epitopes of the B subunit of El Tor and classical biotype *Vibrio cholerae* O1 cholera toxin. J Gen Microbiol 135: 1195–1200. 2482860 10.1099/00221287-135-5-1195

[42] Marchlewicz BA, Finkelstein RA (1983) Immunological differences among the cholera/coli family of enterotoxins. Diagn Microbiol Infect Dis 1: 129–138. 6201315 10.1016/0732-8893(83)90042-1

[43] Fazil MH, Kumar S, Farmer R, Pandey HP, Singh DV (2011) Binding efficiencies of carbohydrate ligands with different genotypes of cholera toxin B: molecular modeling, dynamics and docking simulation studies. J Mol Model. 10.1007/s00894-010-0947-6 21409571

[44] Ansaruzzaman M, Bhuiyan NA, Nair BG, Sack DA, Lucas M, et al (2004) Cholera in Mozambique, variant of *Vibrio cholerae* . Emerg Infect Dis 10: 2057–2059. 16010751 10.3201/eid1011.040682 PMC3329043

[45] Nair GB, Faruque SM, Bhuiyan NA, Kamruzzaman M, Siddique AK, et al (2002) New variants of *Vibrio cholerae* O1 biotype El Tor with attributes of the classical biotype from hospitalized patients with acute diarrhea in Bangladesh. J Clin Microbiol 40: 3296–3299. 12202569 10.1128/JCM.40.9.3296-3299.2002 PMC130785

[46] Nair GB, Qadri F, Holmgren J, Svennerholm AM, Safa A, et al (2006) Cholera due to altered El Tor strains of *Vibrio cholerae* O1 in Bangladesh. J Clin Microbiol 44: 4211–4213. 16957040 10.1128/JCM.01304-06 PMC1698305

[47] Raychoudhuri A, Patra T, Ghosh K, Ramamurthy T, Nandy RK, et al (2009) Classical ctxB in *Vibrio cholerae* O1, Kolkata, India. Emerg Infect Dis 15: 131–132. 19116078 10.3201/eid1501.080543 PMC2660696

[48] Gustafsson L, Hallander HO, Olin P, Reizenstein E, Storsaeter J (1996) A controlled trial of a two-component acellular, a five-component acellular, and a whole-cell pertussis vaccine. N Engl J Med 334: 349–355. 8538705 10.1056/NEJM199602083340602

[49] Dertzbaugh MT, Elson CO (1993) Reduction in oral immunogenicity of cholera toxin B subunit by N-terminal peptide addition. Infect Immun 61: 384–390. 8423068 10.1128/iai.61.2.384-390.1993 PMC302741

[50] Reed LJ, Muench H (1938) A simple method of estimating fifty percent endpoints. The American Journal of Hygiene 27: 493–497.

